# Supplementary material for: Pressure vessel-oriented visual inspection method based on deep learning
Source: PLoS One. 2022 May 2;17(5):e0267743. doi: 10.1371/journal.pone.0267743 (PMC9060373; doi:10.1371/journal.pone.0267743)
Supplement: Attachment — Submitted filename: Response to Editor and Reviewer Comments.docx [file pone.0267743.s001.docx]

Respones to Editor and Reviewer

Dear Editor and Reviewer:

Thank you for your letter and for the reviewers’ comments concerning our manuscript entitled “Pressure vessel-oriented visual inspection method based on deep learning” (ID: PONE-D-22-00457). Those comments are all valuable and very helpful for revising and improving our paper, as well as the important guiding significance to our researches. We have studied comments carefully and have made correction which we hope meet with approval. Revised portion are marked with “Track Changes” in the paper. The main corrections in the paper and the responds to the reviewer’s comments are as flowing:

**Journal Requirements:**

When submitting your revision, we need you to address these additional requirements.

**1:** Please ensure that your manuscript meets PLOS ONE's style requirements, including those for file naming.

**Response 1: According to the editor’s suggestion, we edit our manuscript to meets PLOS ONE's style requirements, including those for file naming.**

**2:** We suggest you thoroughly copyedit your manuscript for language usage, spelling, and grammar. If you do not know anyone who can help you do this, you may wish to consider employing a professional scientific editing service.

**Response 2: Thank you for your suggestions. I have used Editage to edit my manuscript for language usage, spelling, and grammar. The editing certificate is in the attachment.**

**3:** Please note that PLOS ONE has specific guidelines on code sharing for submissions in which author-generated code underpins the findings in the manuscript. In these cases, all author-generated code must be made available without restrictions upon publication of the work. Please review our guidelines at https://journals.plos.org/plosone/s/materials-and-software-sharing#loc-sharing-code and ensure that your code is shared in a way that follows best practice and facilitates reproducibility and reuse.

**Response 3: According to the editor’s suggestion, I have uploaded my code in https://gitee.com/Meliao/CAD.**

**4:** We note that the grant information you provided in the ‘Funding Information’ and ‘Financial Disclosure’ sections do not match.

**Response 4: Thank you for the reminder, ‘Financial Disclosure’ modify to the following: This work was supported by the Science and Technology Plan Project of the State Administration for Market Regulation, grant number 2019MK143.**

**5:** We note that you have stated that you will provide repository information for your data at acceptance. Should your manuscript be accepted for publication, we will hold it until you provide the relevant accession numbers or DOIs necessary to access your data. If you wish to make changes to your Data Availability statement, please describe these changes in your cover letter and we will update your Data Availability statement to reflect the information you provide.

**Response 5: According to the editor’s suggestion, I have added Data Availability statement in my manuscript.**

**6:** We note that you have indicated that data from this study are available upon request. PLOS only allows data to be available upon request if there are legal or ethical restrictions on sharing data publicly.

**Response 6: According to the editor’s suggestion, I have uploaded data to the website https://gitee.com/Meliao/CAD.**

**7:** Please review your reference list to ensure that it is complete and correct. If you have cited papers that have been retracted, please include the rationale for doing so in the manuscript text, or remove these references and replace them with relevant current references.

**Response 7: According to the editor’s suggestion, I have updated my reference list.**

**Reviewer #1:**

**1:** This study presented by the authors is well organized, the presentation of the article and the results are close to satisfactory. However, the reviewed literature studies are not up-to-date, the article should be reupload by examining the recent studies (especially 2020- 2021).

**Response 1: Thank you for your suggestions.** **I have added the last 2 years of research in the introduction section.**

**Attachment**
